# Concatenation of Human Connexin26 (hCx26) and Human Connexin46 (hCx46) for the Analysis of Heteromeric Gap Junction Hemichannels and Heterotypic Gap Junction Channels

**DOI:** 10.3390/ijms19092742

**Published:** 2018-09-13

**Authors:** Patrik Schadzek, Doris Hermes, Yannick Stahl, Nadine Dilger, Anaclet Ngezahayo

**Affiliations:** 1 Institut für Biophysik, Leibniz Universität Hannover, Herrenhäuser Straße 2, 30419 Hannover, Germany; p.schadzek@biophysik.uni-hannover.de (P.S.); hermes@em.mpg.de (D.H.); yannick.stahl1@googlemail.com (Y.S.); n.dilger@biophysik.uni-hannover.de (N.D.); 2 Department of Clinical Neurophysiology, University of Göttingen, Robert-Koch Str. 40, D-37075 Göttingen, Germany; 3 Zentrum für Systemische Neurowissenschaften Stiftung Tierärztliche Hochschule Hannover, Bünteweg 2, 30559 Hannover, Germany

**Keywords:** oligomerization, concatenated connexins, gap junction, channel stoichiometry, heteromeric connexons, human connexin46, human connexin26, inside-out patch-clamp configuration, dual whole-cell patch-clamp, dye transfer

## Abstract

Gap junction channels and hemichannels formed by concatenated connexins were analyzed. Monomeric (hCx26, hCx46), homodimeric (hCx46-hCx46, hCx26-hCx26), and heterodimeric (hCx26-hCx46, hCx46-hCx26) constructs, coupled to GFP, were expressed in HeLa cells. Confocal microscopy showed that the tandems formed gap junction plaques with a reduced plaque area compared to monomeric hCx26 or hCx46. Dye transfer experiments showed that concatenation allows metabolic transfer. Expressed in *Xenopus* oocytes, the inside-out patch-clamp configuration showed single channels with a conductance of about 46 pS and 39 pS for hemichannels composed of hCx46 and hCx26 monomers, respectively, when chloride was replaced by gluconate on both membrane sides. The conductance was reduced for hCx46-hCx46 and hCx26-hCx26 homodimers, probably due to the concatenation. Heteromerized hemichannels, depending on the connexin-order, were characterized by substates at 26 pS and 16 pS for hCx46-hCx26 and 31 pS and 20 pS for hCx26-hCx46. Because of the linker between the connexins, the properties of the formed hemichannels and gap junction channels (e.g., single channel conductance) may not represent the properties of hetero-oligomerized channels. However, should the removal of the linker be successful, this method could be used to analyze the electrical and metabolic selectivity of such channels and the physiological consequences for a tissue.

## 1. Introduction

Gap junction channels (GJC) are formed between adjacent cells by docking of two hemichannels. The hemichannels are formed by oligomerization of connexins (Cx). Connexins are membrane proteins encoded by a gene family which in the human genome comprises 21 members [1]. In many tissues, different connexins are concurrently expressed and the formation of heteromeric connexons and heterotypic gap junction channels has been postulated. It is assumed that by forming heteromeric connexons and heterotypic gap junction channels, the cells in a tissue could achieve a rectification of gap junctions as it is observed in tissue [2,3]. Using expression systems such as the *Xenopus* oocytes, the formation of heterotypic gap junction channels could be unequivocally demonstrated by coupling two oocytes expressing two different connexins [4]. With such experiments, it was possible to specify which connexins were able to form heterotypic gap junction channels with each other. In combination with molecular biology and protein modeling, it was possible to classify the connexins into two different groups depending on some residues found in the second extracellular loop (EL2). One group is represented by Cx26 and named K-N group with the sequence ϕ(K/R)CXXXPCPNXVDCΩψS and a second group is represented by Cx43 called H group with the sequence ϕXCXXXPCPHXVDCΩψS. In the sequences, ϕ represents a hydrophobic residue, X is any residue, Ω an aromatic residue, and ψ indicates a residue with a large aliphatic side chain [5,6,7,8,9,10]. Within a group the connexins formed compatible connexons. An asparagine residue in position 168 of Cx26 or in a homologous position in other connexins belonging to the K-N group was shown to form hydrogen bonds and was therefore essential for the docking between hemichannels of this group [11,12,13]. The analysis of hCx26, for which a crystal structure was generated, revealed that each asparagine residue at position 176 (N176) in a hemichannel formed three hydrogen bonds with a lysine residue at position 168 (K168), a threonine residue at position 177 (T177), and an aspartic acid residue at position 179 (D179) in the E2 domain of the counterpart hCx26 in the hemichannel of the adjacent cell [6,7,8,9,11,14,15,16,17,18]. For hCx32 and hCx46, homologous N residues to N176 were described [12,13,19,20]. For hCx32, the central N residue was N175, which interacted with K167, T176, and D178. For hCx46, the N188 formed corresponding hydrogen bonds with R180, T189, and D191. The importance of N176 and K168 for the docking interaction was recently demonstrated by Karademir et al. (2016) [21]. The authors showed that by adapting the homologous residues, heterotypic docking between Cx26 and Cx40 connexons could be achieved.

With respect to oligomerization in connexons, the first transmembrane domain (TM1) and the transition between the cytoplasmic loop (CL) and the third transmembrane domain (TM3) were identified as the critical regions [10,22,23]. Analyzing Cx26 mutants, the sequence V37-A40 (VVAA) of the wild type Cx26 was identified as an important motive for Cx26 oligomerization. However, as stated in Jara et al. (2012), the motive did not determine hetero-oligomerization of connexins [10,22,23]. Concerning the hetero-oligomerization of different connexin types within a connexon, the compatibility between connexins was mostly related to the amino acid residues in the region of the transition between the cytoplasmic loop (CL) and the third transmembrane domain (TM3) [10]. According to the sequence of these regions, the connexins were classified into the R-type connexins, which contain a conserved arginine or lysine motif in this region, and the W-type connexins with a di-tryptophan motif. In compliance to this classification, only connexins belonging to the same type can hetero-oligomerize. However, even if the motif in this region is important for the oligomerization, it was proposed that indirect mechanisms associated with the motif were necessary to achieve the discrimination between connexins that do not belong to the same type. Cx43 proteins, for example, are maintained as monomers in the endoplasmic reticulum (ER) by the chaperone protein ERp29, which is associated with the second extracellular loop, thereby avoiding oligomerization. After the transport to the trans-Golgi network, the Cx43 proteins are separated from ERp29 for oligomerization [24]. Therefore, it was suggested that the sequence did not per se hinder the oligomerization between connexins belonging to different types. The sequence was rather an element involved in recruiting quality control proteins like ERp29 that in turn regulated the oligomerization.

**Figure 1:**
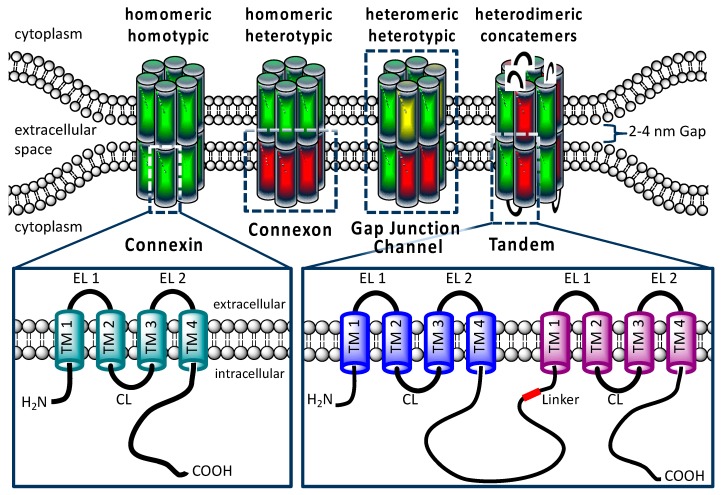
Structural organization of connexins and gap junctions. A gap junction channel is composed of two hemichannels or connexons, which consist of six connexins. Connexins have four transmembrane domains (TM), two extracellular loops (EL), one cytoplasmatic loop (CL), as well an intracellularly localized N- and C-terminus. Homomeric connexons and homotypic gap junction channels are formed by a single connexin isoform. In the postulated heteromeric connexons and heterotypic gap junction channels, different connexin isoforms are expected. The lower right pictogram shows the constructed concatemeric connexins as heterodimeric tandem.

Concatenation of subunits of the GABA, Ach, and P_2×7_ ionotropic receptors has been used to analyse the architecture of these membrane channels [25,26,27,28]. In these studies, it was shown that concatenated proteins were completely inserted in the cell membrane. In the present report, we concatenated hCx26 and hCx46 (Figure 1). Cx26 and Cx46 are concurrently expressed in trachea and alveolar epithelium type 1 and 2 cells [29,30]. With respect to the docking they belong to the K-N group. Therefore, they form hemichannels that can dock to each other. But with respect to the oligomerization behavior, Cx26 belongs to the W-type while Cx46 belongs to the R-type so that they are not supposed to oligomerize [10]. The analysis of the formed gap junction channels with imaging methods and functional assays, as well as electrophysiological characterization, showed that the concatenated proteins were able to form functional hemichannels and gap junction channels. Although aspects of the channels e.g., single channel conductance might be affected by concatenation, aspects such as docking of hetero-oligomerized connexins in variable and clearly determined stoichiometry could be analyzed using concatenation of connexins. For an accurate analysis of the biophysical properties of the concatenated channels, it would be desirable to cleave the linkers of the concatenated connexins in a connexon to reintroduce the full C- and N-terminal flexibility (and thus the natural functionality), as it was done for the concatenated pannexin1 [31].

## 2. Results

### 2.1. Gap Junction Plaques Formed by Concatenated Variants of hCx26 and hCx46

Molecular cloning was used to generate concatenated hCx26-hCx26-GFP (green fluorescent protein), hCx46-hCx46-GFP, hCx26-hCx46-GFP, and hCx46-hCx26-GFP. In order to concatenate two neighboring connexins, a 19-amino acid long linker was inserted into the sequence. The linker, which was used between the connexin and the GFP tag, was 23 amino acids long (Table 1).

**Figure 2:**
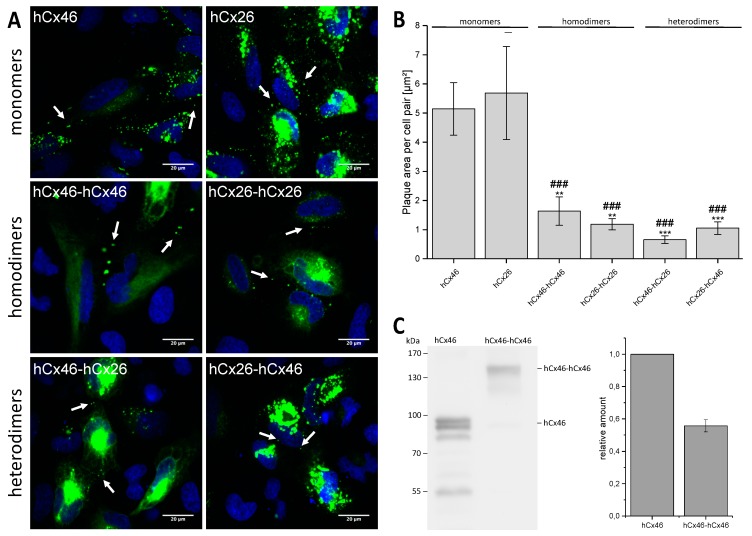
Expression of the GFP-labeled monomeric and concatemeric connexins in HeLa cells. (**A**) Representative micrographs of cell pairs expressing GFP-labeled hCx46, hCx26, hCx46-hCx46, hCx26-hCx26, hCx46-hCx26, and hCx26-hCx46 are shown. The cells were imaged 24 h after transfection using a confocal laser scanning microscope. The nuclei (blue) were stained with Hoechst 33342. Gap junction plaques are indicated by arrows. Gap junction plaques were found in HeLa cells expressing hCx46, hCx26, and the four different tandems. In cells expressing the tandems, a trend to accumulate the proteins in intracellular organelles was observed. (**B**) Quantification of the gap junction plaque area formed by the monomers and the four different tandems in HeLa cells. The plaque area was calculated using the particle analyzer of ImageJ and normalized to the number of transfected cell pairs. At least three transfections were performed per construct. The results are given as average plaque area per cell pair [µm^2^]. Error bars represent the SEM. The data were evaluated by a one-way ANOVA and a post-hoc Tukey test (** *p* ≤ 0.01, *** *p* ≤ 0.001) in comparison to hCx46 and hCx26 (### *p* ≤ 0.001). (**C**) Quantification of the relative protein amount in HeLa cells expressing the monomeric hCx46-GFP or the homodimeric hCx46-hCx46-GFP. An anti-Cx46 antibody was used for the western blotting. For the quantification, four independent replicates were analyzed by using the gel analyzer tool of the FiJi software [32]. The data was normalized to the intensity of the hCx46 monomer.

The concatenated constructs, as well as hCx26-GFP and hCx46-GFP, were expressed in HeLa cells to analyze their capacity to form gap junction plaques. Confocal laser scanning microscopy showed that the GFP labeled constructs formed gap junction plaques between neighboring cells (Figure 2A and Figure S1 in the Supplemental Materials). Compared to the expressed monomeric hCx26-GFP or hCx46-GFP, the dimeric connexins formed gap junction plaques with a reduced surface, suggesting a possible reduction of the protein synthesis for both heterodimeric and homodimeric tandems (Figure 2B). Moreover, western blotting of the hCx46 monomer and hCx46-hCx46 homodimer seems to corroborate the suggestion (Figure 2C). When quantifying the protein amount of the hCx46 monomer and the hCx46-hCx46 homodimer using an anti-Cx46 antibody, cells expressing the homodimer showed a reduction of about 40% of the relative hCx46 amount compared to cells expressing the monomeric hCx46.

**Figure 3:**
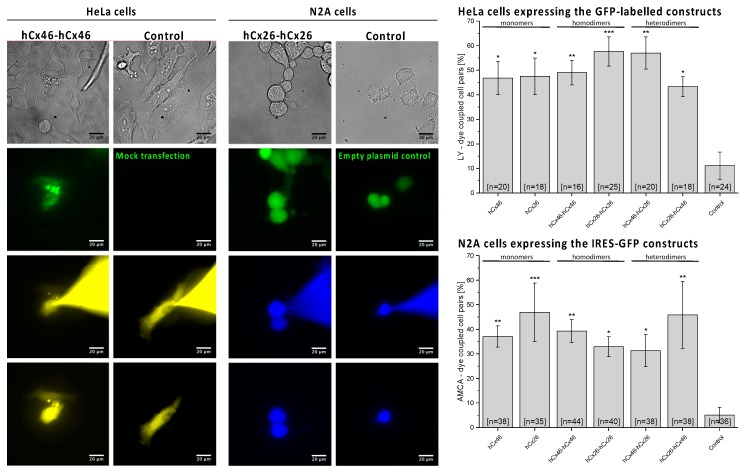
Analyzing the gap junction functionality by dye transfer experiments. A whole-cell patch-clamp configuration with a pipette solution containing 1 mg/mL Lucifer yellow was established on a HeLa cell pair expressing GFP-labelled monomeric hCx26 or hCx46 or one of the concatemeric variants. Mock transfected cells were used as control. The first row of the micrographs shows the phase contrast images of example experiments. In the second row, the GFP fluorescence signal before a dye transfer experiment is shown. The third and fourth rows show the fluorescence signal of the tracer dye 5 min and 10 min after establishment of the whole cell configuration. For the sake of clarity, the image in the fourth row was taken after removal of the dye filled capillary. Likewise, the experiments were performed with N2A cells. The cells were transfected with IRES-GFP constructs resulting in the expression of untagged constructs in the membrane and GFP in the cytosol. As control, cells expressing only GFP were used. The experiments were performed with a pipette solution containing 1 mg/mL AMCA, which could easily be distinguished from GFP under the fluorescence microscope. The cells were considered as coupled if the fluorescence intensity, which was measured in the unpatched cell of a cell pair after 10 min, was at least twice as bright as the background, which was measured at the beginning of the experiment. The probability of coupling (bar diagrams) was estimated as ratio of the sum of coupled cell pairs per the sum of tested pairs. The results are given as average. Error bars represent the SEM. The data were evaluated by a one-way ANOVA and post-hoc Tukey test (* *p* ≤ 0.05, ** *p* ≤ 0.01, *** *p* ≤ 0.01) in comparison to the control cells.

To test the physiological functionality of the built gap junction channels, dye transfer experiments with the monomeric and the tandem connexins in all variations were performed in HeLa and N2A cells (Figure 3). HeLa cells, transfected with either a GFP-labelled monomeric connexin or a homo- or heterodimeric tandem, showed a degree of Lucifer Yellow dye coupling of about 45%, while mock-transfected HeLa cells showed a significantly reduced degree of dye coupling (11%). When using AMCA (7-amino-4-methyl-3-coumarinylacetic acid) as tracer dye in Neuro2A (N2A) cells, expressing the untagged homo- or heterodimeric tandems or the monomeric variants together with soluble GFP (IRES-GFP plasmids), a dye transfer rate between 32% and 46% could be observed, while N2A cells expressing only the soluble GFP showed a significantly reduced dye coupling ability of about 5%. Interestingly, the concatenation did not alter the formation of the functionally coupled gap junction channels.

### 2.2. Single Channel Activity of Connexons

**Figure 4:**
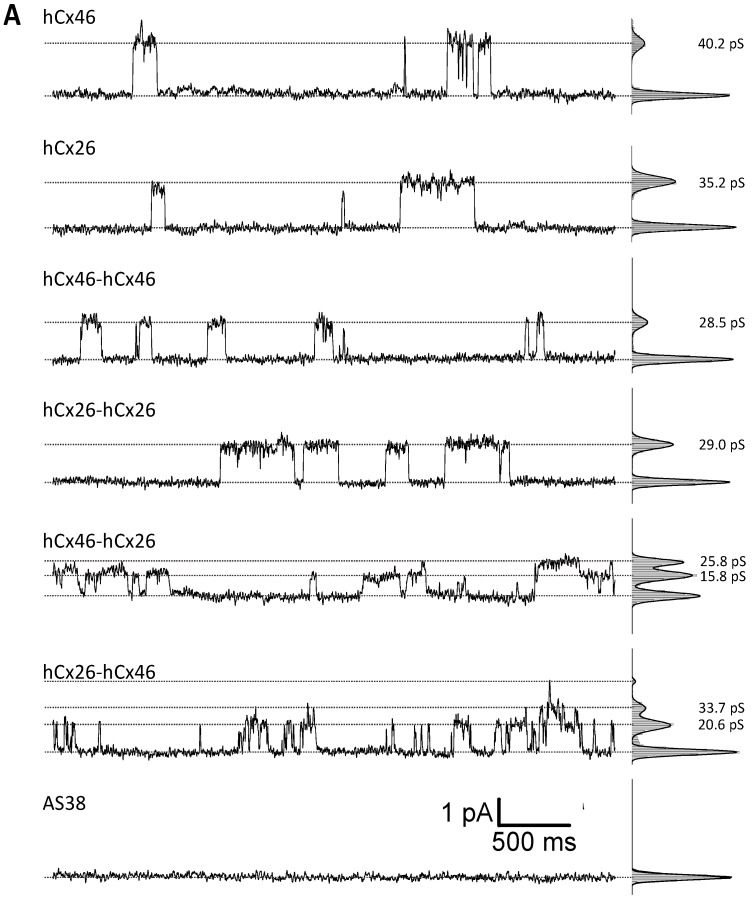

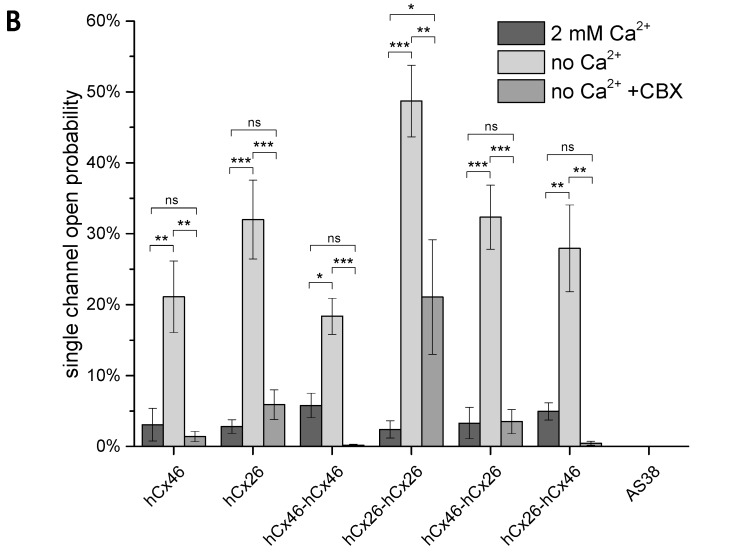
Analysis of single hemichannels formed by concatemeric connexins. The stripped membrane of *Xenopus* oocytes, which were injected with hCx46, hCx26, or the four different concatemeric constructs, as well as the AS38 (control) cRNA 24 h before, was used to perform the inside-out patch-clamp recordings. The measurements were performed in presence of Cs^+^ and in absence of Cl^−^ on both sides of the membrane. (**A**) Examples of single channel currents elicited by a depolarizing voltage pulse of +50 mV in absence of Ca^2+^ in the bath solution are shown. (**B**) The open probability of all measured single channels was analyzed. The error bars represent the SEM. The data were evaluated by a one-way ANOVA followed by a Tukey test (* *p* ≤ 0.05, ** *p* ≤ 0.01, *** *p* ≤ 0.001, ns: not significant). The statistical comparison showed that the presence of Ca^2+^ or carbenoxolone (CBX) in the bath solution significantly reduced the open probability of all tested variants.

Cx46 is known to form gap junction hemichannels when expressed in *Xenopus* oocytes [33,34,35]. Therefore, *Xenopus* oocytes were used as expression system to measure the single channel activity of connexons formed by the single connexins, as well as the variant tandems. In inside-out patch-clamp experiments, we found that the open probability *p* of channels composed of the monomeric connexins, as well as the homodimers and heterodimers, was increased by suppression of Ca^2+^ on both side of the channels (Figure 4). Moreover, we observed that all configurations were sensitive to the gap junction channel inhibitor carbenoxolone (CBX). With respect to CBX, the Cx26 connexons were less sensitive to the agent than the hCx46 connexons (Figure 4). The insensitivity to CBX was even more pronounced for the hCx26-hCx26 homodimer, while the hCx46-hCx46 heterodimeric hemichannels were almost completely closed by CBX (Figure 4B). For the hemichannels formed by the heterodimers in either configuration, the sensitivity to CBX was more similar to that observed for hCx46 connexons than that of hCx26 connexons. For a further characterization of the hemichannels, we analyzed the single channel conductance in absence of Ca^2+^ (Figure 4A). Under our experimental conditions, in which the chloride was completely replaced by gluconate at both sides of the membrane, a single channel conductance of 46.0 ± 5.3 pS and 39.2 ± 5.5 pS was found for hCx46 and hCx26, respectively. For the homodimers, the conductance was reduced to 24.5 ± 3.3 pS and 32.9 ± 6.3 pS for hCx46-hCx46 and hCx26-hCx26, respectively. For the heterodimers, two substates were observed for each configuration: A conductance of 15.7 ± 0.8 pS and 26.2 ± 1.8 pS for hCx46-hCx26, and 20.1 ± 1.4 pS and 31.2 ± 3.0 pS for the hCx26-hCx46 configuration (Table 2, Figure 4). The changes in conductance are also related to the concatenation. A successful cleavage of the linker to separate the connexins in the channels is needed in order to evaluate changes solely caused by hetero-oligomerization of the connexins within a channel.

### 2.3. Dye Uptake through Hemichannels

**Figure 5:**
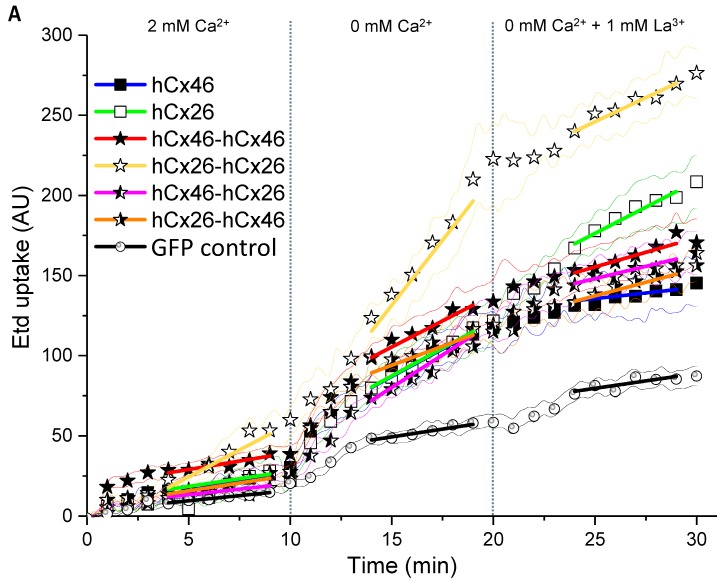

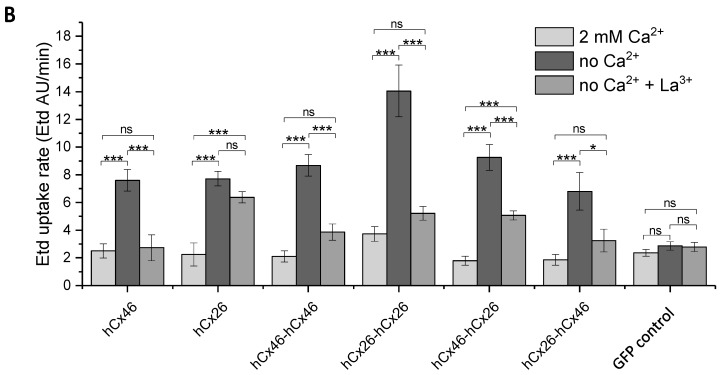
Dye uptake through hemichannels using ethidium bromide. HeLa cells were grown on coverslips to a confluency of about 40–50% and transfected with IRES-GFP-plasmids. The GFP allowed the identification of transfected cells by fluorescent microscopy. (**A**) Time course of dye uptake experiments by cells expressing the different variants when perfused with bath solutions containing 2 mM Ca^2+^, no Ca^2+^, and no Ca^2+^ but 1 mM La^3+^. The fine lines show the SEM spread for all measured points. The symbols indicate the average for data points measured every 1 min. The solid lines indicate the part of the curves that was used to estimate the dye uptake rate. (**B**) Quantification of the dye uptake rate (Etd AU/min) for all tested variants and the backbone control in absence or presence of Ca^2+^ or La^3+^. The error bars represent the SEM. The data were evaluated by a one-way ANOVA and a post-hoc Tukey test (* *p* ≤ 0.05, *** *p* ≤ 0.001, ns: not significant).

Using ethidium bromide (Etd), we observed the dye uptake by HeLa cells expressing hemichannels formed by the different variants in order to clarify how the constructed tandems could affect the function of the channels. First, we found that the cells expressing the monomers or the tandems in different variations did not differ from mock cells in their capacity to absorb ethidium bromide as long as external Ca^2+^ was present (Figure 5). The dye uptake in presence of external Ca^2+^ was therefore considered as background uptake. Specific hemichannel uptake of the dye was initialized when the cells were superfused with a Ca^2+^-free external solution (Figure 5). To determine a possible mechanical effect on the dye uptake, the cells were first superfused with a 2 mM Ca^2+^-containing solution. In cells expressing the homodimeric hCx26-hCx26, a tendency to increase the rate of dye uptake during the perfusion with the Ca^2+^-containing solution was observed. However, this mechanical sensitivity of the channel was not statistically significant when analyzed by a two-way ANOVA and a post-hoc Tukey test. For cells expressing the GFP control or the other variants, the tendency to respond to a mechanical stimulus was not observed (Figure 5). For all variants, the ethidium bromide uptake was accelerated by the superfusion of the cells with Ca^2+^-free external solution compared to cells expressing the GFP control (Figure 5A,B and Figure S2: Table in the Supplemental Materials). In the context of low external Ca^2+^, only the hCx26-hCx26 homodimer showed an increased rate of dye uptake compared to the monomeric hCx26 and hCx46, as well as the hCx46-hCx46 homodimer and both heterodimers (S2). These changes in the ethidium bromide dye uptake rate seems to be an artifact due to the concatenation. For hCx46 however, the hCx46-hCx46 homodimer as well as the hCx46 monomer showed a comparable rate of dye uptake, and this uptake rate of hCx46 was also measured for the heterodimers in either order. Additionally, dye uptake by hemichannels in all variants except for the hCx26 monomer (and the GFP control cells) was significantly reduced by the superfusion with a La^3+^-containing medium. When using a two-way ANOVA and a post-hoc Tukey test for the comparison of the different variants, neither the perfusion with the Ca^2+^-containing solution nor the perfusion with a Ca^2+^-free and La^3+^-containing solution showed a significant difference to another variant or the GFP expressing control cells. Only the perfusion with the Ca^2+^-free solution led to significant differences, as described above (S2).

### 2.4. Activity of Single Gap Junction Channels

**Figure 6:**
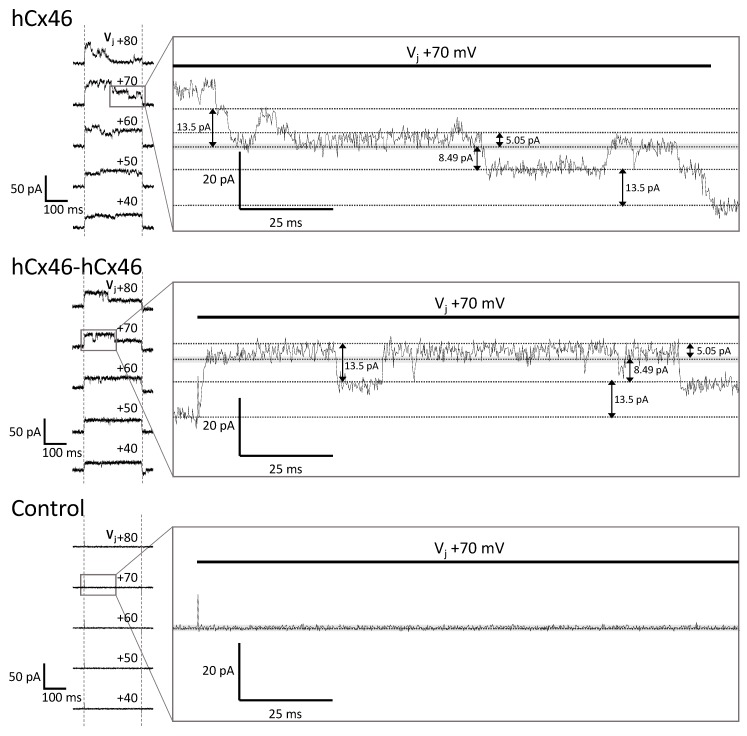
Recordings of dual whole-cell patch-clamp experiments. N2A cells were cultured and transfected with the different IRES-GFP-plasmids. As control, cells expressing only soluble GFP in the cytosol were used. Twenty-four hours post transfection, the dual whole-cell patch-clamp experiments were performed. The resting membrane potential was set to −40 mV for both cells. One cell of a cell pair was alternatingly stepped from −120 mV to +60 mV, while the junctional currents were recorded in the other cell. The junctional currents (ΔI2) recorded during the 250 ms-long voltage pulses at different transjunctional potentials are shown above the current responses. Magnification of the Vj +70 mV traces of the hCx46 monomer, as well as of the hCx46-hCx46 homodimer, showed several simultaneously open channels, with a large conductance of ~193 pS (13.5 pA step), and low conductance of ~72 pS (5.05 pA step) and ~121 pS (8.49 pA step). Similar steps were observed for Cx46 by other authors [38,39,40,41]. The control cells showed only the background noise, which was below 2 pA (grey band), indicating that the fluctuations of about 5 pA were conducting substates.

The activity of single gap junction channels was analyzed by dual whole-cell patch-clamp experiments [36,37] applied on N2A cells expressing hCx26 and hCx46, as well as the different dimeric variants. Compared to HeLa cells, which were used for the analysis of gap junction plaques, N2A cells did not form a monolayer, which offered the advantage of an easy identification of unambiguous cell pairs suitable for the dual whole-cell patch-clamp experiments. Moreover, probably due to their round morphology, the cells formed small gap junction plaques with less gap junction channels allowing a better observation of single gap junction channel activity (Figure 6).

Cells expressing the different variants were found to form gap junction channels with a total macroscopic conductance of up to 1000 pS. In some of these cell pairs, it was possible to follow the closing and opening of single gap junction channels at different transjunctional voltages, and to estimate the conductance of the single gap junction channels in dependency of the expressed variant. Although multiple simultaneously opened channels were recorded, it was possible to follow the opening and closing of single gap junction channels (Figure 6).

Considering the clear opening and closing of the single channels, maximal conductance levels of 202 pS, 198 pS, 138 pS, 184 pS, 137 pS, and 371 pS were estimated for the Cx46 monomer, Cx46-Cx46 homodimer, Cx26 monomer, Cx26-Cx26 homodimer, Cx46-Cx26 heterodimer, and Cx26-Cx46 heterodimer, respectively. The mean of the large conductance ± SEM of these variants is given in Table 3. Besides these large conductance levels, low subconductance states were observed (Figure 6 and Figure S3 in the Supplemental Materials). However, because of the rapid flickering, a clear estimation of the numeric values was not possible.

## 3. Discussion

In cells, different connexin isoforms are concurrently expressed. The formation of heteromeric connexons and heterotypic channels with a variable stoichiometry offers the cell a mode for fine tuning the gap junction coupling, thereby producing a flux rectification and selectivity [3,4]. The goal of the present report was to test whether concatenated connexins form heteromeric connexons and heterotypic gap junction channels with clearly determined stoichiometry (Figure 1). This could be a promising method to study the physiological consequences of the hetero-oligomerization of connexins in hemichannels and gap junction channels.

By expressing GFP labeled hCx46 and hCx26 monomers, hCx46-hCx46 and hCx26-hCx26 homodimers, as well as hCx46-hCx26 and hCx26-hCx46 heterodimers in HeLa cells, we found that the monomers as well as the homodimers and heterodimers were transported to the cell membrane and were able to form gap junction plaques (Figure 2A). However, as shown for the hCx46 monomer and the hCx46-hCx46 homodimer, the concatenation reduced the quantity of the produced protein. This might contribute to the reduction of the gap junction plaque area formed by the tandems (Figure 2B). The results suggest that concatenation might induce changes in the synthesis or the trafficking of the proteins to the membrane. Furthermore, a tendency to retain the proteins in the ER and Golgi apparatus was observed in cells expressing the hCx46-hCx46 tandem compared to cells expressing the hCx46 monomer (Figure 2; cells expressing the hCx26 per se showed a higher intracellular signal, which limited the possibility to compare trafficking of homomers and dimers). Although this trend was not statistically significant, it could indicate a possible concatenation-related problem with protein trafficking. Nevertheless, the different tandem proteins as well as the monomers formed functional gap junction channels, as demonstrated by the dye transfer experiments (Figure 3). Consequently, the concatenation of connexins might be helpful for the analysis of hetero-oligomerization of connexins and, inter alia, opens the possibility to prospectively study metabolite selectivity, which could not be predicted so far. Oligomerization, trafficking to the membrane, and the assembly to gap junction plaques were not affected when GFP was coupled to the N-terminus. However, the fusion of a fluorescent protein tag to the N-terminus of a connexin has been shown to block the channel conductance of hemichannels and gap junction channels [42,43]. The mechanism responsible for this is still a matter of speculation. The crystal structure of Cx26 led to the prediction that the N-termini folded in the pore of the channel [11]. We can assume that the hydrophilic GFP subunits coupled to the N-termini might stay in the cytosol. They might hinder the correct folding of the N-terminus or form a lid on the cytosolic mouth of the channel. In case of the concatenated connexins, the hydrophobic transmembrane domains of the two linked connexins led to the formation of a protein with eight transmembrane domains. How the connexin C-terminus is structured is not fully understood. However, experimental data showed that the C-terminus is highly flexible [44] and interacts with cytoskeleton-associated proteins to structure the gap junction plaques in the cell membrane, suggesting that the C-terminus does not form a barrel like GFP [45]. We therefore assume that, by interacting with these associated proteins and in combination with the high flexibility, the C-terminus of the first connexin of the concatemer could be close enough to the membrane to allow a correct structure formation of the linked N-terminus of the following connexin. However, the NMR (nuclear magnetic resonance) data also showed disorders in the C-termini that can be affected by binding to partners. Additionally, the NMR data showed the capability of dimerization in some parts of the C-termini [44]. Consequently, it is possible that the concatenation, even if it is compatible with the formation of hemichannels and gap junction channels, may affect the properties of the channels e.g., single channel conductance. Therefore, concatenation offers the possibility to form hemichannels with a determined stoichiometry and gap junction channels with manageable variabilities. However, a tool for the removal of the linker between the connexins after formation of the hemichannels is still needed to allow an exploitation of the potentialities of the method.

Considering the single channel conductance, the inside-out patch-clamp analysis of connexons formed in the membrane of *Xenopus* oocytes showed conductance values of about 46 pS and 39 pS for hCx46 and hCx26 monomers, respectively. In isolated lens fibers (Cx46) of rodents, a conductance of about 240 pS was measured [46]. Similarly, for Cx46 hemichannels expressed in *Xenopus* oocytes, HeLa, or N2A cells, main conductance levels of 250–300 pS could be identified [47,48,49,50]. For Cx26 expressed in *Xenopus* oocytes, HeLa, or N2A cells, a main conductance of 320 pS [51] and even a higher conductance above 400 pS was found [52]. In our inside-out patch-clamp experiments, we could not identify these large conductance values. However, the conductance values presented in this report of about 39 pS and 46 pS for hemichannels composed of hCx26 and hCx46 monomers are similar to measured subconductance states of these channels that were published by other authors [38,39,40,41]. For Cx26, Gaßmann et al. (2009) reported three conductance states with G_1_ = 34 ± 8 pS, G_2_ = 70 ± 8 pS, and G_3_ = 165 ± 19 pS. G_1_ represents the vast majority of the detected events [39]. Moreover, the conductance values were measured after the replacement of chloride by the less mobile gluconate and acetate ions on both sides of the membrane. This allowed silencing the background currents and thereby isolating specific currents that were only observed in patches from oocytes expressing connexons (Figure 4). It is known that the replacement of chloride by less mobile ions leads to lower conductance values [53,54]. We therefore assume that the measurement of hemichannels formed by hCx46 and hCx26 monomers in our experimental conditions, where chloride was replaced by gluconate and acetate ions, only allowed the recording of substates with a low conductance.

The conductance values measured for the hCx46-hCx46 (24.5 ± 3.3 pS) and hCx26-hCx26 (32.9 ± 6.3 pS) homodimers were slightly lower than the conductance values found for the hemichannels formed by the respective monomers. Although the shown differences are comparably slight, concatenation-related artifacts might need to be suppressed by successfully removing the linker between the proteins within the hemichannels to reveal the properties of heteromerization-related changes. However, by allowing the generation of channels with a defined stoichiometry, concatenation could allow studying the aspects of heterodimerization of connexins. Correspondingly, we found that heterodimerization of hCx26 and hCx46 introduced two new substates with ~16 pS and ~26 pS for hCx46-hCx26, and ~20 pS and ~31 pS for hCx26-hCx46. The conductance of hemichannels formed by hCx46-hCx46 or hCx26-hCx26 homodimers was clearly different to that of hemichannels formed by hCx46-hCx26 or hCx26-hCx46 heterodimers, respectively. This result suggests that both parts of the concatenated connexins participated in the hemichannels. Further non-published data showed that concatenation of the hCx46 and the hCx46N188T mutant, which did not form gap junction plaques [13], reduced the formation of gap junction plaques compared to the homodimer hCx46-hCx46, suggesting that in concatenated form the hCx46N188T participated to formation of gap junction channels. The observation that concatenated connexins are inserted in the membrane as whole is an agreement with other experiments in which subunits of membrane proteins such as Ach, GABA, or ATP ionotropic receptors channels were concatenated [25,26,27,28]. As for the changes in conductance observed in the present report, the results indicate that the formation of heterodimeric hemichannels would change the conductance and opening properties of the channels in comparison to the respective homomeric hemichannels. A separation of the two connexins in a concatemer is desirable for the analysis of the biophysical properties of the channel.

Dual whole-cell patch-clamp experiments were performed to analyze the corresponding gap junction channels. The hCx46 and hCx26 homomers formed gap junction channels with a maximal conductance of ~200 pS for hCx46 and ~140 pS for hCx26. In other expression systems, similar conductance values were found for the gap junction channels formed by these connexins [41,55]. Beside these main conductance levels, other open substates and other residual substates with conductance values of about 20 pS for Cx46 and 17 pS for hCx26 were observed, suggesting that the conductance values (40 pS for hCx46 and 35 pS for hCx26) observed in the membrane of oocytes might represent the residual substates of the hemichannels. The concatenation of hCx46 did not change the maximal conductance of the channels (Figure 6). For hCx26, the concatenation resulted in channels with a slightly increased maximal conductance of about 180 pS. Similar to the hemichannels, the analysis of gap junction channels formed by the heterodimers revealed changes in the channel properties compared to the homodimers. Therefore, these changes, such as the strong increase of the maximal conductance, might be more related to heteromerization than to the concatenation. To confirm this hypothesis, a cleavage of the linker between the connexins in a concatemer is necessary.

The comparison of the electrophysiological data obtained from hCx46-hCx26 and hCx26-hCx46 heterodimers showed some unexpected variability with respect to hemichannels and gap junction channel activity, as well as to conductance states. The hCx26-hCx46 showed a higher activity as hemichannels and as gap junction channels in comparison to homodimers. In the case of gap junction channels, we found that channels formed by hCx26-hCx46 had a more elevated conductance (371 pS) than the channels composed of hCx46-hCx26 (137 pS). It shows that the concatenation might affect some properties of the channels, especially the electrical conductance, in a way that we cannot explain. We presume that this might be related to the short C-terminus of Cx26, which might affect the following N-terminus of Cx46. In their model, Maeda et al. (2009) suggested that the N-termini form a voltage-sensitive funnel in the cytoplasmic mouth of the pore [11]. In our experiments with the tandems, three N-termini were free while the other three termini were linked to the C-termini of the preceding molecule. If the C-terminus of Cx46 is linked, the length of this terminus may allow more flexibility to the linked N-terminus than if the C-terminus is given by Cx26, which is very short. The observation that the homodimer hCx26-hCx26 formed hemichannels and gap junction channels with more activity, as well as gap junction channels with higher conductance levels than those of the Cx26 homomers, is compatible with this presumption. Additionally, the presumed model could explain the difference in the trend of insensitivity to the inhibition by La^3+^. Using ethidium bromide, Jara et al. (2012) already showed a degree of insensitivity of Cx26 hemichannels to La^3+^ [22]. This insensitivity was transferred to the hemichannels formed by hCx46-hCx26 heteromers and did not affect the hCx26-hCx46 heteromers (Figure 5). This trend might be related to the number of free N-termini. Because the C-terminus of Cx46 in hCx46-hCx26 is long, the N-terminus of the following hCx26 has more freedom to move. As result, we have three N-termini of hCx26, which are almost free to interact with the completely free N-termini of hCx46. In hCx26-hCx46, three N-termini of hCx46 are linked to the short C-termini of hCx26, thereby limiting the interaction of these N-termini with the free N-termini of hCx26. At that point, the concatenation limits the bearing of the information shown in this report. However, it will be a valuable method if the cleavage of the linker is successful. An increase of the conductance would be a good indication of a successful removal of the linker.

Using different methods, in this report we showed that concatenation could be a technique to understand the consequences of formation of heteromeric gap junction hemichannels, as well as of heterotypic gap junction channels built between cells. Concerning the oligomerization of connexins, two critical motifs, which were not mutually exclusive, have been identified: The end of the first transmembrane domain (TM1) and the transition between the cytoplasmic loop (CL) and the third transmembrane domain (TM3) [10,22,23]. The motif in TM1 which was found to be critical for the oligomerization of Cx26 was not important for hetero-oligomerization [10,22,23]. On the other side, the amino acid dendrogram showed that the compatibility of different connexin isoforms to hetero-oligomerize into a connexon was related to a motif situated at the transition region between CL and TM3 [10]. According to the sequence of these regions, the connexins were classified between the R-type connexins, which contain a conserved arginine or lysine residue, and the W-type connexins with a di-tryptophan motif. According to this classification, connexins belonging to different types do not oligomerize.

In our experiments, we showed that Cx26 (W-type) and Cx46 (R-type), which are also different in the TM1 region, could form heteromeric connexons with each other. Thereby, our results support the assumption that the control of hetero-oligomerization is not regulate by the TM1 [10,22,23]. Moreover, the residues R and W of the connexins in the transition between the cytoplasmic loop (CL) and the third transmembrane domain (TM3) are indirect control and not an intrinsic property of the connexins that regulate the hetero-oligomerization. Our results show the importance of indirect controls for hetero-oligomerization above the sequences in the different parts of the connexins.

## 4. Materials and Methods

### 4.1. Molecular Cloning

In order to express various concatemers in HeLa cells, as well as in N2A cells and in *Xenopus laevis* oocytes, the multisite gateway cloning system with three different destination plasmids was used.

For the transfection of the cell lines, the destination plasmids pEF-I-GFP GX, which has an IRES element between the gateway cassette and the reporter GFP, and the psDEST47 were used. pEF-I-GFP GX [56] was a gift from John Brigande (Addgene plasmid # 45443). psDEST47 was created by using a “reverse” BP-cloning reaction with the expression clone pcDNA-DEST47-GFP-GFP and the pDONR221 linearized with EcoNI. pcDNA-DEST47-GFP-GFP [57] was a gift from Patrick Van Oostveldt (Addgene plasmid # 36139). The psDEST47 was transformed into ccdB survival *Escherichia coli* BD3.1 cells and selected on ampicillin- and chloramphenicol-containing LB-Agar plates. The purified psDEST47, which is similar to the commercially available pcDNA™-DEST47 vector (#12281010, Thermo Fisher Scientific, Waltham, MA, USA), was used to create a C-terminally GFP-labeled fusion protein via the LR-cloning reaction.

The vector psGEMHE-GW was used for in vitro transcription to produce the cRNA for the *Xenopus* oocytes. The vector was created by restriction enzyme cloning with XbaI and HindIII using the pGEMHE [35] as backbone. As insert the gateway cassette was amplified with the attR1-ccdB-attR2 XbaI F and attR1-ccdB-attR2 HindIII R primers (see Table 4). *Escherichia coli* BD3.1 cells (invitrogen, Carlsbad, CA, USA) were used to host the three different destination vectors.

The various Entry vectors were built by amplifying hCx46 or hCx26 with the primers listed in Table 4 with a proofreading DNA polymerase (Phusion, Thermo Fisher Scientific, Waltham, MA, USA) followed by the BP-clonase reaction (Thermo Fisher Scientific, Waltham, MA, USA) with the donor plasmids pDONR™221, pDONR™221 P1-P5r, and pDONR™221 P5-P2 (Thermo Fisher Scientific, Waltham, MA, USA). For the psGEMHE-GW and the pEF-I-GFP GX plasmids, the stop attB2 R primers were used. *Escherichia coli* MachI (Thermo Fisher Scientific, Waltham, MA, USA) was used to host the ten different Entry plasmids. The purified Entry and destination plasmids were used to perform the multisite LR reaction (LR clonase II plus, Thermo Fisher Scientific, Waltham, MA, USA). *Escherichia coli* MachI was used to host the 18 different expression clones (three different destination plasmids, each with monomeric hCx46 and hCx26, homodimeric hCx46-hCx46 and hCx26-hCx26, as well as the heterodimeric hCx46-hCx26 and hCx26-hCx46). The BP-clonase II and LR-clonase II plus reactions were successfully performed in a total volume of only 2.5 µL. Restriction enzyme cloning and gateway cloning were verified by sequencing (Seqlab, Göttingen, Germany).

### 4.2. Cell Culture

HeLa cells (DSMZ no.: ACC 57, Leibniz Institute DSMZ-German Collection of Microorganisms and Cell Cultures, Braunschweig, Germany) were cultured in DMEM/Ham’s F12 (1:1) medium (FG 4815, Biochrom, Berlin, Germany) supplemented with 10% fetal calf serum (Biochrom), 1 mg/mL penicillin, and 0.1 mg/mL streptomycin (Biochrom). The mouse neuroblastoma cells N2A, abbreviation for Neuro-2A (DSMZ no.: ACC 148), were cultured in DMEM with 1.0 g/L D-glucose (FG 0415, Biochrom) supplemented with 10% heat inactivated fetal calf serum (Biochrom), 1x non-essential amino acids (Biochrom), 1 mg/mL penicillin, and 0.1 mg/mL streptomycin (Biochrom). The cells were cultured in a humidified atmosphere with 5% CO_2_ at 37 °C. Every two to three days the cell culture medium was renewed.

### 4.3. Quantification of the Expression Behavior

To analyze the formation of gap junctions, 7 × 10^4^ HeLa cells were seeded on collagen I-coated glass coverslips with a diameter of 1 cm into a well of a 24-well plate 24 h before transfection to reach a confluency of about 70–80%. Prior to the transfection, the cell culture medium was replaced by 500 µL OptiMEM I medium (Thermo Fisher Scientific). The transfection was performed as described before [13,58,59]. In brief, per well, 500 ng purified plasmid and 1.5 µL FuGene HD (Promega, Mannheim, Germany) transfection reagent were incubated in 25 µL OptiMEM I medium for 15 min at room temperature and added to the prepared cells. After 4–6 h, the transfection medium was exchanged to the penicillin- and streptomycin-free culture medium.

For the quantification of the expression behavior, the psDEST47 constructs were used, which resulted in C-terminally labeled GFP fusion proteins. The cells were fixed 24 h after transfection with 3.7% formaldehyde. The nuclei of the cells were stained with Hoechst 33342 (1 µg/mL; Sigma Aldrich, St. Louis, MO, USA) and the cell membranes were stained with Alexa 555-conjungated Wheat Germ Agglutinin (5 µg/mL; Molecular Probes, Eugene, OR, USA) to improve the visibility of the cell-cell contact regions. The cells were imaged with a confocal Nikon Eclipse TE2000-E C1 laser scanning microscope (Nikon, Düsseldorf, Germany) as described previously [13,58,59]. For each variant, at least five different transfections and coverslips were evaluated. Four images were taken of different regions of each coverslip.

To analyze the plaque area per cell pair, the micrographs were evaluated using FiJi [32]. The resulting plaque areas per cell pair of the concatemers were evaluated in comparison to the monomeric hCx46 and hCx26 by using a one-way ANOVA, followed by a Tukey test and are given as mean ± SEM.

### 4.4. Western Blot

HeLa cells were grown to about 80% confluence in a 100 mm diameter cell culture plate. The cells were transfected with the psDEST47 hCx46 or the psDEST47 hCx46-hCx46 plasmids and cultivated for further 24 h. For the transfection of a 100 mm diameter cell culture plate, 5 µg plasmid DNA and 15 µL FuGene HD were used (details are described above in Section 4.3). For the protein isolation, the cells were washed twice with ice-cold PBS and were removed from the culture plate with a cell scraper in presence of 1 mL ice-cold PBS. After a centrifugation step at 750× *g* for 3 min at 4 °C, the pellet was resuspended in 50 µL RIPA buffer containing 25 mM Tris HCl pH 7.6, 150 mM NaCl, 1% nonidet P-40, 1% sodium desoxycholate, 0.1% SDS, freshly added 0.5% protease inhibitor cocktail (Roche, Waiblingen, Germany), 10 mM NaF, 1 mM PMSF, and 1 mM Na_3_VO_4_. After an incubation for 15 min on ice, a centrifugation at 14,000× *g* for 15 min at 4 °C was used to separate the protein solution from the cell debris. A Bradford assay (Sigma Aldrich) was used to determine the protein concentration of the supernatant using BSA as standard. 1 × Laemmli buffer (13 mM Tris HCl, 10 mM DTT, 2% glycerol, 0.4% SDS, 0.002% Bromphenol Blue, pH 6.8) was added to the protein solution and incubated for 10 min at 70 °C. Next, 100 µg protein per lane were separated in a 5% SDS-polyacrylamide stacking gel and a 10% separation gel. The proteins were transferred to a nitrocellulose membrane using a semi-dry blot (transfer buffer: 25 mM Tris HCL, pH 8.3, 192 mM glycine, 0.1% SDS, and 20% methanol). Afterwards, the membrane was blocked with 5% non-fat dry milk powder in PBS containing 0.1% Tween 20 (PBS-T) for 2 h at room temperature. Anti-Cx46 antibody (sc-365394, Santa Cruz Biotechnology, Heidelberg, Germany) was diluted 1:1000 in PBS-T and applied to the membranes for an overnight incubation at 4 °C. After washing, the secondary anti-mouse antibody (A9044, Sigma Aldrich) was diluted 1:100,000 and applied for 1 h at room temperature. For the detection, the SuperSignal West chemiluminescent reagent (Thermo Fisher Scientific) was used. The blot was imaged with a CCD camera system (Intas Science Imaging, Göttingen, Germany). For the quantification, four independent replicates were analyzed by using the gel analyzer tool of the FiJi software [32]. Data are displayed normalized to the intensity of the hCx46 monomer.

### 4.5. Dye Transfer Experiments

To test the functionality of the formed gap junction channels, dye transfer experiments with Lucifer Yellow and 7-amino-4-methyl-3-coumarinylacetic acid (AMCA) in HeLa and N2A cells were performed, respectively. HeLa cells were prepared and transfected with the different psDEST47-plasmids as described above in Section 4.3. As control, mock transfected HeLa cells were used. For the dye transfer experiments with the N2A cells, the cells were transfected with the different pEF-I-GFP GX-plasmids. For control experiments, the N2A cells were transfected with the empty destination vector pEF-I-GFP GX. Coverslips with transfected cells were transferred to a perfusion chamber containing 400 µL of a bath medium consisting of (in mM) 140 NaCl, 5 KCl, 10 HEPES, 10 glucose, 1 MgCl_2_, and 2 CaCl_2_ at pH 7.4 and osmolarity (π) of 295 mosmol/L. The chamber was mounted on an inverted fluorescence microscope (Ti-E, Nikon GmbH, Duesseldorf, Germany) equipped with a Polychrome V monochromator (T.I.L.L. Photonics GmbH, Planegg, Germany), a CCD Orca-Flash 4.0 camera (Hamamatsu Photonics Deutschland GmbH, Herrsching, Germany), and the NIS-Elements AR 4.4 software (Nikon GmbH).

For the dye transfer experiments, a whole-cell patch-clamp configuration was established on one cell of a transfected cell pair using an EPC 10 USB double patch-clamp amplifier (HEKA Elektronik Dr. Schulze GmbH, Lambrecht/Pfalz, Germany) coupled to the PatchMaster 2.9 software (HEKA Elektronik Dr. Schulze GmbH). For the pipette filling solution used for the HeLa cells, 1 mg/mL Lucifer Yellow (LY) lithium salt (Biotium, Hayward, CA, USA) was diluted in a pipette medium containing (in mM) 145 K gluconate, 5 KCl, 10 HEPES, 2.5 MgATP, 5 glucose, 0.5 Na_2_ATP, 1 EGTA, and 0.2 CaCl_2_ at pH 7.4 and π 295 mosmol/L. For the experiments with N2A cells, the LY was replaced by 1 mg/mL AMCA (Sigma Aldrich, St. Louis, MO, USA). The Polychrome V was used to excite the GFP-labeled connexin variants at 488 nm, LY at 410 nm, and AMCA at 350 nm. For each dye transfer experiment, micrographs of the GFP and LY or AMCA fluorescence were taken before the whole-cell configuration was established, during the experiment, and after 10 min with prior removal of the LY or AMCA containing pipette. For each variant, the degree of dye coupling was estimated as the ratio of the number of coupled pairs to the total number of tested pairs expressing the particular variant. The results are given as mean values ± SEM. The significance of the difference was evaluated by a one-way ANOVA and a post-hoc Tukey test (*** for *p* ≤ 0.001, ** for *p* ≤ 0.01, and * for *p* ≤ 0.05).

### 4.6. Expression in Xenopus Oocytes

For the in vitro transcription, the mMESSAGE mMACHINE^®^ T7 (Thermo Fisher Scientific, Waltham, MA, USA) and the PeaI-linearized (Thermo Fisher Scientific) psGEMHE vectors were used to generate the artificial cRNA. The cRNA was purified by a phenol/chloroform extraction and an isopropanol precipitation.

*Xenopus laevis* oocytes were harvested from an anaesthetized female frog. After mechanical disruption of the tissue, the oocytes were separated by an incubation (1 h at room temperature) in 190–240 U/mL collagenase type II (Worthington, Berlin, Germany) containing oocyte control medium composed of (in mM) 88 NaCl, 1 KCl, 10 Tris-HCl, and 0.82 MgCl_2_ (pH 7.4 and π 180 mosmol/L). During the tissue digestion with collagenase, the tissue was shaken at 100 rpm. After the collagenase treatment, the oocytes were washed with oocyte control medium supplemented with 2 mM CaCl_2_. The oocytes were stored and used for injection for up to three days after isolation.

Stage V and stage VI oocytes were injected with 23 nL of an aqueous solution containing 1 µg/µL connexin mRNA and 400 ng/µL antisense to the endogenous Cx38 (AS38) using the Nanoliter Injector (World Precision Instruments, Berlin, Germany). The antisense DNA (AS38) with the sequence C*T*GACTGCTCGTCTGTCCACAC*A*G* (* indicates phosphorothioate modification) was purchased from Microsynth AG (Balgach, Switzerland). The injected oocytes were incubated at 16 °C in the Ca^2+^-containing oocyte medium and used for the measurement of single channels 18 to 48 h post injection.

### 4.7. Single Channel Recordings of the Connexons

For the recording of the single channels, the vitelline membrane of a connexin expressing oocyte was mechanically removed. The oocyte was incubated for at least 3 min in a stripping solution containing (in mM) 88 NaCl, 1 KCl, 10 Tris-HCl, 0.82 MgCl_2_, 2 CaCl_2_, and 200 D-mannitol (pH 7.4 and π 444 mosmol/L) to release the vitelline membrane from the oocyte membrane. The released vitelline membrane was removed using two Dumont no. 5 forceps (Manufactures D’Outils Dumont SA, Montignez, Switzerland) under a LEICA GZ4 binocular (Leica Mikrossysteme Vertrieb GmbH, Wetzlar, Germany). The stripped oocyte was transferred into a perfusion chamber containing 400 µL of a control bath solution consisting of (in mM) 88 Na gluconate, 1 K gluconate, 10 Tris, 2 Ca acetate, 0.82 Mg acetate, and 20 Cs acetate (pH 7.4 and π 220 mosmol/L) and mounted on an inverted fluorescence microscope described in Section 4.4. The patch-clamp experiments were performed using an EPC 10 USB double patch-clamp amplifier (HEKA Elektronik Dr. Schulze GmbH). The data were recorded with filter 1 (Bessel) at 10 kHz and filter 2 (I_Bessel) at 1 kHz. The patch pipettes were made from PG150T-7.5 glass capillaries (Clark Electromedical Instruments, Pangbourne, UK). Filled with the pipette filling solution composed of (in mM) 80 Na gluconate, 20 Cs acetate, and 10 HEPES (pH 7.4 and π 220 mosmol/L) the pipettes had an electrical resistance of about 5 MΩ. The reference electrode was filled with K gluconate solution to avoid problems related to Ag/AgCl junction [60].

Three minutes after the formation of a gigaseal, the inside-out patch-clamp configuration [61] was established. To measure the single channels, test voltage pulses between −70 mV and 60 mV were applied for 20 s in 10 mV steps. Between the voltage pulses, the membrane was clamped at 0 mV for 30 s. Thereafter, the bath solution was changed to a Ca^2+^-free solution composed of (in mM) 88 Na gluconate, 1 K gluconate, 10 Tris, 0.82 Mg acetate, and 20 Cs acetate at pH 7.4 and π 220 mosmol/L. After application of the test voltages, the membrane was perfused with a Ca^2+^-free solution supplied with 100 µM carbenoxolone (CBX) and the voltage pulses were applied again.

For the data analysis and the example curves, the data were filtered with the digital filter at 100 Hz in the FitMaster 2.90 software (HEKA Elektronik Dr. Schulze GmbH). The software was also used to generate an amplitude histogram, which was fitted using a multi Gaussian fit to calculate the single channel conductance states. The single channel open probability was estimated by using the single channel event detection tool of the FitMaster software. Simultaneous opening of channels was rarely observed. If it was observed this event was excluded in the calculation of the single channel open probability. For the calculation, only the +40 mV to +60 mV traces, which had an unambiguous signal-to-noise ratio, were used. A measurement time of at least 4 min for at least four injected oocytes of each variant was used for the estimation. For the comparison of the data a one-way ANOVA followed by a Tukey test was used.

### 4.8. Dye Uptake through Hemichannels

The hemichannel activity was analyzed by measuring the ethidium bromide (Etd) uptake slightly modified from Schalper et al. (2008) [62]. A day before the experiment, subconfluent HeLa cells grown on collagen I-coated coverslips were transfected with the pEF-I-GFP variants. For the control group experiments, the empty destination plasmid pEF-I-GFP GX was used to transfect the cells. A coverslip with transfected cells was placed in a perfusion chamber with a chamber volume of approximately 400 µL mounted on an inverse Nikon Ti-E fluorescence microscope, as described in Section 4.4. The ISMATEC REGIO ICC peristaltic pump (Cole-Parmer GmbH, Wertheim, Germany) controlled by the software ISMATEC^®^ Pump Control (Cole-Parmer GmbH) allowed the constant exchange of the medium with a flow rate of 1 mL/min. Prior to the experiment, the GFP fluorescence of the transfected cells was used to define the regions of interest (ROIs) for the Etd uptake measurement.

During the first 10 min of a 30-min long dye uptake experiment, the cells in the chamber were perfused with a prewarmed (37 °C) bath solution composed of (in mM) 121 NaCl, 5.4 KCl, 25 HEPES, 0.8 MgCl_2_, 5.5 glucose, 6 NaHCO_3_, 2 CaCl_2_, and 5 µM ethidium bromide (pH 7.4 and π 296 mosmol/L). After 10 min, the medium was exchanged for additional 10 min to a Ca^2+^- and Mg^2+^-free solution, which was consisting of (in mM) 121 NaCl, 5.4 KCl, 25 HEPES, 5.5 glucose, 6 NaHCO_3_, and 5 µM ethidium bromide (pH 7.4 and π 295 mosmol/L). In the last 10 min of a dye uptake experiment, 1 mM La^3+^ was added to the Ca^2+^/Mg^2+^-free solution. Before starting an experiment, regions of interest (ROIs) were selected in a fluorescent micrograph of the cells taken by an Orca flash 4.0 CCD camera (Hamamatsu Photonics Germany, Herrsching am Ammersee, Germany). During the entire experiment, fluorescent images were taken every 15 s with an exposure time of 700 ms. The images were used to assess the changes of the fluorescence intensity of the ROIs during an experiment. For the recording of the images and the measurement of fluorescence intensity in the ROIs, the NIS-Elements AR 4.4 software (Nikon GmbH) was used. The dye uptake rate (Etd AU/min) was calculated with OriginPro 2017 (OriginLab Corporation, Northampton, MA, USA) from minute 4–9, 14–19, and 24–29, respectively (stationary rate). The results are given as mean values ± SEM. The significance of the difference was evaluated by a one-way ANOVA and a post-hoc Tukey test (*** for *p* ≤ 0.001, ** for *p* ≤ 0.01, and * for *p* ≤ 0.05). For the comparison between the groups a two-way ANOVA followed by a Tukey test was used.

### 4.9. Dual Whole-Cell Patch-Clamp Experiments

For the dual whole-cell patch-clamp experiments, approximately 3 × 10^4^ N2A cells were cultured on a collagen I-coated coverslip in a well of a 24-well plate and were transfected as described above with the pEF-I-GFP variants, which resulted in the separate expression of the different connexin-variants and GFP as the reporter. For the control experiments, the N2A cells were transfected with the empty pEF-I-GFP destination vector. The coverslip with the transfected cells was transferred into a perfusion chamber filled with 400 µL of a bath solution composed of (in mM) 121 NaCl, 5.4 KCl, 25 HEPES, 0.8 MgCl_2_, 5.5 glucose, 6 NaHCO_3_, 2 CaCl_2_ and mounted on the inverse Nikon Ti-E fluorescence microscope described in Section 4.4. The patch pipettes were filled with a pipette solution containing (in mM) 125 K gluconate, 15 CsCl, 0.2 CaCl_2_, 2.5 MgCl_2_, 1 MgATP, 5 glucose, 0.5 EGTA, 4 Na_2_ATP, 0.1 cAMP, and 10 HEPES (pH 7.4 and π 295 mosmol/L). The patch pipettes were made from 40A502 glass capillaries (Kimble Chase Life Science and Research Products, Rockwood, TN, USA). Filled with the pipette solution the pipettes had an electrical resistance of 2–5 MΩ.

Dual whole-cell patch-clamp experiments were performed with the EPC 10 USB double patch-clamp amplifier described in Section 4.6. After establishing a whole-cell configuration on both cells of a cell pair, both cells were clamped at −40 mV. For the measurements, one cell of the cell pair (cell 1) was alternatingly stepped from −120 mV to +60 mV (V_1_) for a duration of 250 ms, while the junctional currents were recorded in the other cell (cell 2), which was maintained at −40 mV (V_2_). The transjunctional voltage gradient (V_j_ = V_2_ − V_1_) was calculated.

## 5. Conclusions

In summary, the present paper shows that the expression of concatenated connexins leads to a reduced plaque area between cells. However, concatenation of connexins was compatible with trafficking of the hemichannels to the membrane and the formation of functional gap junction hemichannels in the cell membrane and gap junction channels between cells. It could be used to generate hemichannels and gap junction channels with a determined stoichiometry. Because of the linker between the connexins, the properties of the formed hemichannels and gap junction channels (e.g., single channel conductance) do not represent the properties of hetero-oligomerized channels. However, should the removal of the linker be successful, this method could be used to analyze the electrical and metabolic selectivity of such channels and the physiological consequences for a tissue.

## Figures and Tables

**Table 1:** The amino acid linkers (one-letter code) between the two concatenated connexins and between the connexin and the GFP tag. The GFP tag was always located at the C-terminus.

Linker	Amino Acid Sequence
Cx-Cx	GGNLQSTVPR ATTLYTKVV
Cx-GFP	GGNLQSTVPR AHPAFLYKVV RSR

**Table 2:** Conductance states of the hemichannels expressed in *Xenopus* oocytes as measured in inside out patch-clamp configuration. n gives the number of analyzed oocytes.

Injected cRNA	Large Substates ± SEM	Small Substates ± SEM
hCx46 (n = 5)	46.0 ± 5.3 pS	
hCx26 (n = 5)	39.2 ± 5.5 pS	
hCx46-hCx46 (n = 4)	24.5 ± 3.3 pS	
hCx26-hCx26 (n = 7)	32.9 ± 6.3 pS	
hCx46-hCx26 (n = 4)	26.2 ± 1.8 pS	15.7 ± 0.8 pS
hCx26-hCx46 (n = 5)	31.2 ± 3.0 pS	20.1 ± 1.4 pS
AS38 (n = 6)	0 pS	

**Table 3:** Large conductance states of the gap junction channels expressed in transfected N2A cell pairs. n gives the number of analyzed cell pairs for each variant.

Expressed Variant	Large Conductance ± SEM
hCx46 (n = 7)	175.5 ± 5.7 pS
hCx26 (n = 7)	182.8 ± 1.0 pS
hCx46-hCx46 (n = 14)	193.5 ± 2.4 pS
hCx26-hCx26 (n = 9)	125.1 ± 4.0 pS
hCx46-hCx26 (n = 18)	110.5 ± 8.5 pS
hCx26-hCx46 (n = 11)	281.2 ± 24.7 pS
GFP control (n = 8)	0 pS

**Table 4:** Primers used for restriction enzyme cloning to produce the destination vector psGEMHE-GW and for the BP-cloning to generate the various Entry clones.

Primer	5’-3’ Sequence
attR1-ccdB-attR2 XbaI F	CTTCATCTAGACACGCTCGAGATCACAAGTTTGTAC
attR1-ccdB-attR2 HindIII R	CTTCGAAGCTTTTACATCTCGAGCACCACTTTGTACAAG
GW_BP-cloning hCx46 attB1 F	GGGGACAAGTTTGTACAAAAAAGCAGGCTCCATGGGCGACTGGAGCTTTCTGG
GW_BP-cloning hCx46 attB2 R	GGGGACCACTTTGTACAAGAAAGCTGGGTGGGCCCGCGGTACCGTCGAC
GW_BP-cl. hCx46 stop attB2 R	GGGGACCACTTTGTACAAGAAAGCTGGGTTCTAGATGGCCAAGTCCTCCGGT
GW_BP-cloning hCx46 attB5r R	GGGGACAACTTTTGTATACAAAGTTGTGGCCCGCGGTACCGTCG
GW_BP-cloning hCx46 attB5 F	GGGGACAACTTTGTATACAAAAGTTGTAATGGGCGACTGGAGCTTTCTGG
GW_BP-cloning hCx26 attB1 F	GGGGACAAGTTTGTACAAAAAAGCAGGCTTAATGGATTGGGGCACGCT
GW_BP-cloning hCx26 attB2 R	GGGGACCACTTTGTACAAGAAAGCTGGGTTGGCCCGCGGTACCG
GW_BP-cl. hCx26 stop attB2 R	GGGGACCACTTTGTACAAGAAAGCTGGGTTCTAAACTGGCTTTTTTGACTTCCCAGAAC
GW_BP-cloning hCx26 attB5r R	GGGGACAACTTTTGTATACAAAGTTGTGGCCCGCGGTACCG
GW_BP-cloning hCx26 attB5 F	GGGGACAACTTTGTATACAAAAGTTGTAATGGATTGGGGCACGCT

## Supplementary Materials

The following are available online at http://www.mdpi.com/1422-0067/19/9/2742/s1.

## Abbreviations

ACh	acetylcholine
AMCA	7-amino-4-methyl-3-coumarinylacetic acid
ANOVA	analysis of variance
AS38	antisence38 oligonucleotide, against *Xenopus laevis* Cx38
CBX	carbenoxolone
CL	cytoplasmatic loop
Cx	connexin
EL1	first extracellular loop
EL2	second extracellular loop
ER	endoplasmatic reticulum
Etd	ethidium bromide
GABA	*γ*-aminobutyric acid
GJC	gap junction channel
hCx	human connexin
LY	lucifer Yellow
N2A	neuro-2A, mouse neuroblastoma cell line
ns	not significant
ROIs	regions of interest
SEM	standard error of the mean
TM	transmembrane domain
