# Supplementary material for: Concatenation of Human Connexin26 (hCx26) and Human Connexin46 (hCx46) for the Analysis of Heteromeric Gap Junction Hemichannels and Heterotypic Gap Junction Channels
Source: Int J Mol Sci. 2018 Sep 13;19(9):2742. doi: 10.3390/ijms19092742 (PMC6163895; doi:10.3390/ijms19092742)
Supplement: Supplementary file 1 [file ijms-19-02742-s001.pdf]

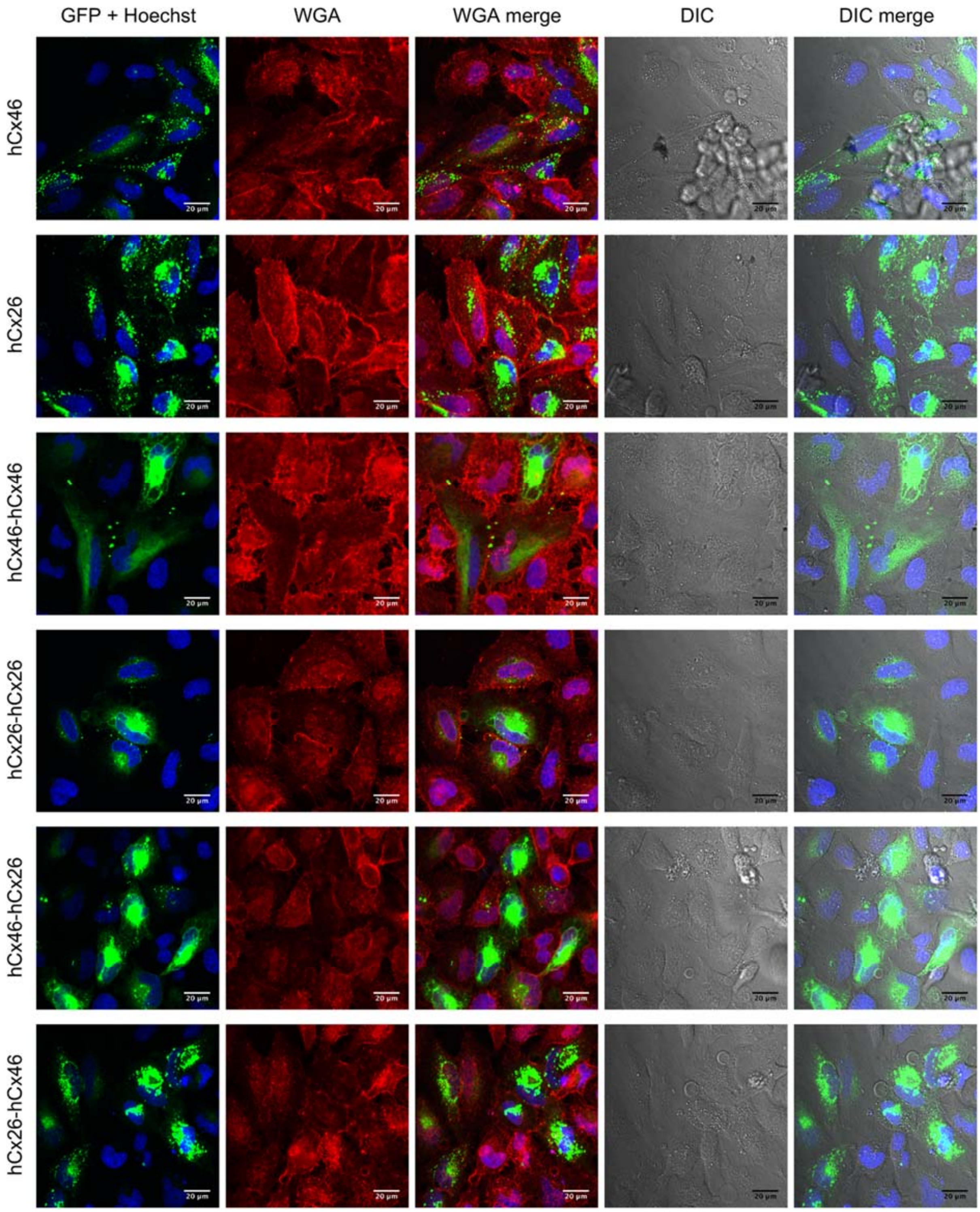

S1: Expression of the eGFP-labeled monomeric and concatemeric connexins in HeLa cells. Representative micrographs of cell pairs expressing eGFP-labeled hCx46, hCx26, hCx46-hCx46, hCx26-hCx26, hCx46-hCx26 and hCx26-hCx46 are shown. The cells were fixed 24 h after transfection with 3.7 % formaldehyde. The nuclei of the cells were stained with Hoechst 33342 (blue) and the cell membranes were stained with Alexa 555-conjungated Wheat Germ Agglutinin (red) to improve the visibility of the cell-cell contact regions. The cells were imaged using a confocal laser scanning microscope. Gap junction plaques were found in HeLa cells expressing hCx46, hCx26 and the four different tandems.

A

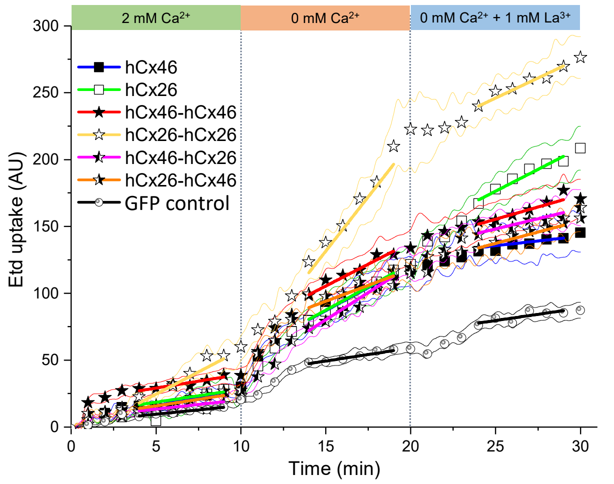

B

2 mM Ca2+	hCx46	hCx26	hCx46-hCx46	hCx26-hCx26	hCx46-hCx26	hCx26-hCx46	GFP
hCx46	x	ns	ns	ns	ns	ns	ns
hCx26		x	ns	ns	ns	ns	ns
hCx46-hCx46			x	ns	ns	ns	ns
hCx26-hCx26				x	ns	ns	ns
hCx46-hCx26					x	ns	ns
hCx26-hCx46						x	ns
GFP							x

no Ca2+	hCx46	hCx26	hCx46-hCx46	hCx26-hCx26	hCx46-hCx26	hCx26-hCx46	GFP
hCx46	x	ns	ns	***	ns	ns	**
hCx26		x	ns	***	ns	ns	*
hCx46-hCx46			x	***	ns	ns	***
hCx26-hCx26				x	***	***	***
hCx46-hCx26					x	ns	***
hCx26-hCx46						x	*
GFP							x

no Ca2+ + 1 mM La3+	hCx46	hCx26	hCx46-hCx46	hCx26-hCx26	hCx46-hCx26	hCx26-hCx46	GFP
hCx46	x	ns	ns	ns	ns	ns	ns
hCx26		x	ns	ns	ns	ns	ns
hCx46-hCx46			x	ns	ns	ns	ns
hCx26-hCx26				x	ns	ns	ns
hCx46-hCx26					x	ns	ns
hCx26-hCx46						x	ns
GFP							x

S2: Comparison of dye uptake of cells expressing the different variants or the GFP control for each perfusion solution. (A) Time course of the dye uptake. The fine lines show the SEM spread for all measured points. The symbols indicate the average for data points measured every 1 min. The solid lines indicate the part of the curves that was used to estimate the dye uptake rate.The fine lines show the SEM of the dye uptake for each variant. The stars indicate the average for each variant. The solid lines indicate the part of the curves that was used to estimate the dye uptake rate. **(B)** Statistical comparison of the dye uptake rate in cells expressing the different variants under consideration of the perfused external solution. A two-way ANOVA and a post-hoc Tukey test was performed for the statistical comparison. * $p \leq 0.05$, ** $p \leq 0.01$, *** $p \leq 0.01$, ns: not significant.

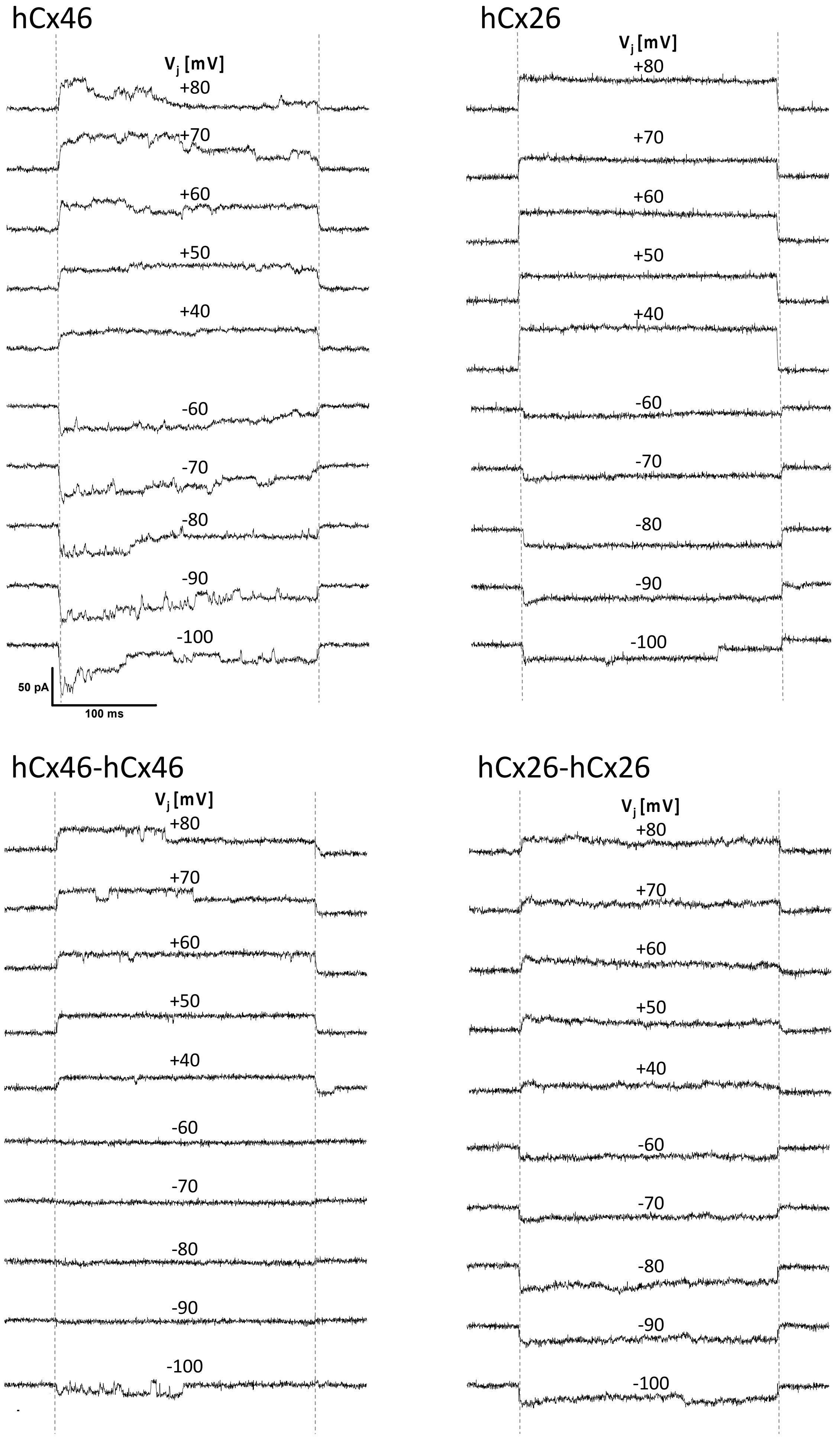
hCx46
Vj [mV]
+80
+70
+60
+50
+40
-60
-70
-80
-90
-100
50 pA
100 ms
hCx26
Vj [mV]
+80
+70
+60
+50
+40
-60
-70
-80
-90
-100
hCx46-hCx46
Vj [mV]
+80
+70
+60
+50
+40
-60
-70
-80
-90
-100
hCx26-hCx26
Vj [mV]
+80
+70
+60
+50
+40
-60
-70
-80
-90
-100

hCx46-hCx26

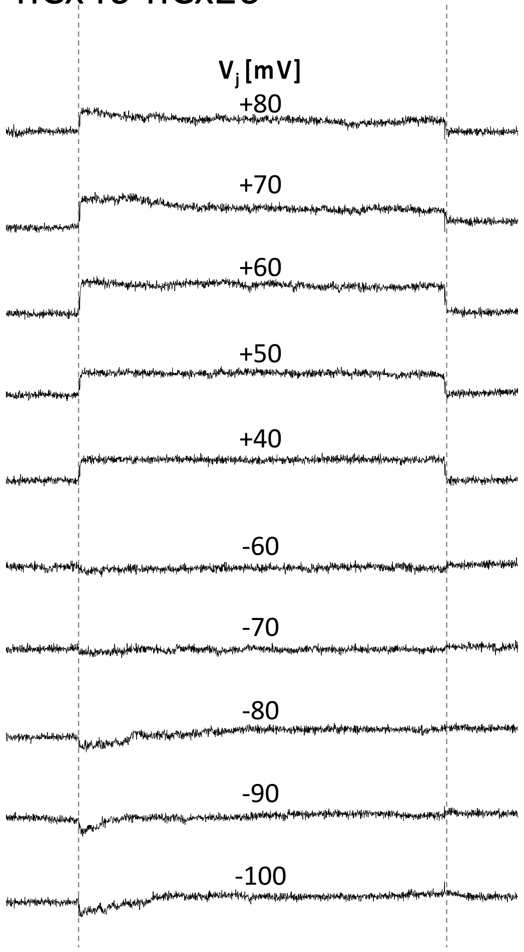

hCx26-hCx46

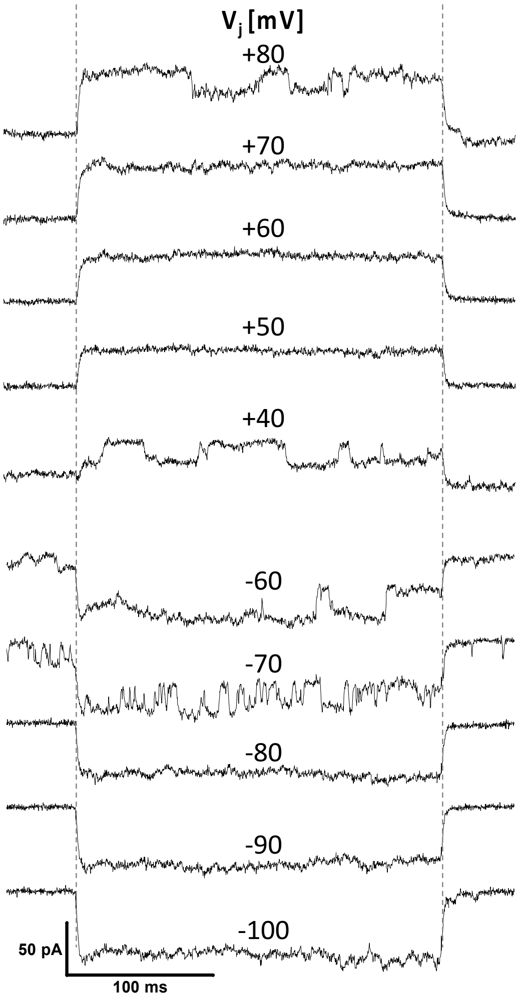

vector control

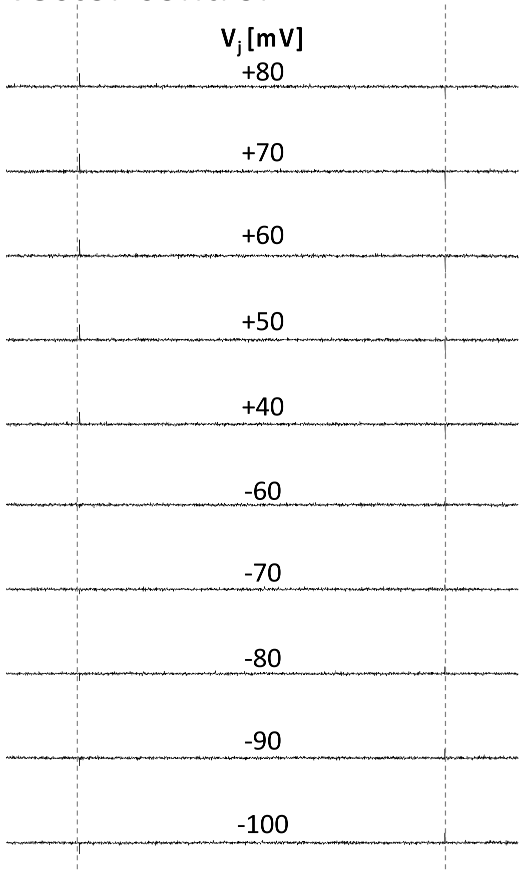

S3: Recordings from the double whole cell patch clamp experiments. N2A cells were cultured and transfected with different IRES-GFP-plasmids. 24 h post transfection the double whole cell patch clamp experiments were performed. The holding potential was set to -40 mV for both cells. Voltage pulses from -120 mV to +60 mV (V_1) were applied in 10 mV steps to one cell of the cell pair (cell 1) while the junctional currents were recorded in the other cell (cell 2), which was maintained at -40 mV (V_2). This induced transjunctional potential pulses ($V_j = V_2 – V_1$) from -100 mV to +80 mV. The recording are the current responses to the indicated V_j.
